# Population Structure and Genetic Diversity among Isolates of *Coccidioides posadasii* in Venezuela and Surrounding Regions

**DOI:** 10.1128/mBio.01976-19

**Published:** 2019-11-26

**Authors:** Marcus M. Teixeira, Primavera Alvarado, Chandler C. Roe, George R. Thompson, José S. L. Patané, Jason W. Sahl, Paul Keim, John N. Galgiani, Anastasia P. Litvintseva, Daniel R. Matute, Bridget M. Barker

**Affiliations:** a The Translational Genomics Research Institute (TGen)—Affiliate of City of Hope, Flagstaff, Arizona, USA; b Pathogen and Microbiome Institute, Northern Arizona University, Flagstaff, Arizona, USA; c Núcleo de Medicina Tropical, Faculdade de Medicina, Universidade de Brasília, Brasília, Brazil; d Servicio Autonomo Instituto de Biomedicina Dr. Jacinto Convit, Caracas, Venezuela; e Departments of Medicine, Division of Infectious Diseases, and Medical Microbiology and Immunology, UC Davis Medical Center, Sacramento, California, USA; f Laboratório Especial de Ciclo Celular, Instituto Butantan, São Paulo, Brazil; g Valley Fever Center for Excellence, University of Arizona College of Medicine, Tucson, Arizona, USA; h Mycotic Diseases Branch, Centers for Disease Control and Prevention, Atlanta, Georgia, USA; i Department of Biology, University of North Carolina, Chapel Hill, North Carolina, USA; Vanderbilt University; Tel Aviv University

**Keywords:** coccidioidomycosis, Venezuela, *Coccidioides posadasii*, Caribbean, Valley Fever

## Abstract

*Coccidioides posadasii* is a pathogenic fungus that causes coccidioidomycosis in many arid regions of the Americas. One of these regions is bordered by the Caribbean Sea, and the surrounding landscape may play an important role in the dispersion of *C. posadasii* across South America through southeastern Mexico, Honduras, Guatemala, and Venezuela. Comparative phylogenomic analyses of *C. posadasii* reveal that clinical strains from Venezuela are genetically distinct from the North American populations found in (i) Arizona and (ii) Texas, Mexico, and the rest of South America (TX/MX/SA). We find evidence for admixture between the Venezuela and the North American populations of *C. posadasii* in Central America. Additionally, the proportion of Venezuelan alleles in the admixed population decreases as latitude (and distance from Venezuela) increases. Our results indicate that the population in Venezuela may have been subjected to a recent bottleneck and shows a strong population structure. This analysis provides insight into potential for *Coccidioides* spp. to invade new regions.

## Abstract (precis)

Valley Fever is a fungal disease caused by two species of fungi: Coccidioides immitis and *C. posadasii*. These fungi are found throughout the arid regions of North and South America; however, our understanding of genetic diversity and disease in South America is limited. In this report, we analyze 10 new genomes of *Coccidioides posadasii* from regions bordering the Caribbean Sea. We show that these populations are distinct and that isolates from Venezuela are likely a result of a recent bottleneck. These data point to patterns that might be observed when investigating recently established populations.

## INTRODUCTION

In spite of their human health impact, fungal pathogens are currently understudied (1, 2). To date, approximately 150 fungal species are responsible for disease in humans, with an estimated 1.5 million deaths each year (3, 4). The most affected are immunocompromised patients (e.g., HIV), but several of these diseases affect otherwise healthy patients (5, 6). Systematic surveys of genetic diversity of fungal pathogens have revealed extensive variability in the strength of virulence among genotypes of isolates from the same species (e.g., reference 7). Other traits like antifungal resistance and the ability to survive different environmental conditions also show extensive variation (8–10). Defining the magnitude and sources of variation among these traits is a crucial aspect of understanding why some pathogens are more effective at spreading and causing disease than others.

An important aspect of variation is not only the total magnitude within a species but also how that variation is apportioned across populations (11, 12). Distinct populations within species and barriers to gene flow can maintain genetic variation within and between species boundaries, respectively. Studying the origin and maintenance of genetic variation is important because it is likely to dictate the pathogen’s ability to cause disease and respond to treatment (13). The paucity of studies that have focused efforts to understand the magnitude of this variation is particularly acute for fungal pathogens that occur in the tropics and subtropics. The tropics are generally more diverse than temperate areas in terms of numbers of species (14–16); thus, the genetic diversity of fungi, and in particular of fungi with the ability to cause disease in humans, might be larger in the tropics as well.

Coccidioidomycosis, or “Valley Fever,” is an example of one of these primary fungal diseases, and is caused by Coccidioides immitis and Coccidioides posadasii (17). The disease range overlaps arid regions throughout the American continent, but California and Arizona encompass the vast majority of reported cases of the disease (18). Due to the availability of clinical isolates, most sampling has occurred in these two U.S. states, even though the geographic range extends into Latin America. Coccidioidomycosis has been reported less frequently in arid and semiarid environments of Central America (Guatemala and Honduras) and South America (Brazil, Argentina, Paraguay, Colombia, and Venezuela) (19, 20). It is unclear if isolates of *Coccidioides* from the southwestern United States show a pattern of higher infectivity and/or virulence, or if this reflects a lower prevalence of the organism in other regions of endemicity in the Americas coupled with lower population densities in most arid regions. Alternatively, the pattern could be explained by lower rates of fungal disease awareness, testing, and reporting from tropical regions.

The two etiological agents of coccidioidomycosis, *C. immitis* and *C. posadasii*, are sibling species that diverged about 5 million years ago (21, 97). The species are thought to be phenotypically similar, but some key differences have been reported. Besides showing a highly differentiated genome, *C. posadasii* grows more slowly than *C. immitis* at high concentrations of salt (22). Additionally, spherules of *C. posadasii* appear to develop asynchronously compared to *C. immitis* (23). There are also broad differences in the geographic distribution of the two species. While *C. immitis* is found predominantly in California, *C. posadasii* has a broader distribution, which ranges from Arizona to Argentina. Consistent with this distribution, coccidioidomycosis in South and Central America is primarily caused by *C. posadasii*, but sporadic reports of *C. immitis* exist from Washington State, Argentina, and Colombia (24–26, 75). Both species of *Coccidioides* show strong signatures of population structure. In the case of *C. immitis*, genomic analyses have revealed the existence of two subdivided populations: Washington State (at the northernmost point of the known range of the species distribution) and the rest of the range (21). In the case of *C. posadasii*, at least two distinct populations have been proposed: (i) Arizona and (ii) Texas, Mexico, and South America (TX/MX/SA) (21, 27).

Studying the population structure of *C. posadasii* is critical to understanding the spread of the pathogen across the Americas. Based on microsatellite analyses, the introduction of *C. posadasii* into South America has been dated to between 9,000 and 140,000 years ago (ya), and was proposed to follow the Amerindian intercontinental migration (28). However, the timing of most recent common ancestor for *C. posadasii* populations, as inferred by whole-genome single nucleotide polymorphism (SNP) analysis, revealed that *C. posadasii* lineages that gave rise to TX/MX/SA and Arizona emerged 700,000 ya (700 kya), and *C. posadasii* Guatemala emerged about 200 kya (21). These two findings are not consistent with a serial bottleneck giving rise to the current South American populations, but are consistent with ancestral population structure previous to the spread of *C. posadasii* to South America and the migration of a few lineages to the south. However, before any of these hypotheses can be tested, an assessment of the genetic characteristics of South American isolates is needed.

One of the most characteristic xeric ecosystems in South America is the Paraguaná xeric shrubland located in Venezuela (29), which is defined by arid and dry climates, low altitude, xerophytic vegetation, and sandy soils with high salt concentration. This environment may favor *Coccidioides* growth and development (30). Notably, skin test surveys using coccidioidin in Latin American communities revealed positive intradermic test rates of 44% and 46% in the Chaco region of Paraguay and the Lara State of Venezuela, respectively (26, 31). Additionally, environmental molecular detection of *Coccidioides posadasii* in Venezuela suggests a high prevalence of the organism in the soil (32). The actual occurrence of coccidioidomycosis remains unclear; as fewer than 1,000 total coccidioidomycosis cases have been reported over the last century in South and Central America (19, 26). However, environmental sampling and serological inquiries suggest that the importance and prevalence of coccidioidomycosis in South America may be vastly underappreciated, and little is known about the genetic characteristics and genealogical relationships of this population.

In this report, we aim to bridge this gap. We sequenced 10 *C. posadasii* isolates and studied their relationship to 72 previously sequenced isolates. Seven Venezuelan isolates form a monophyletic group with little diversity, which is differentiated from other *C. posadasii* populations. Notably, we find that Central American populations of *C. posadasii* are the result of admixture between North America and Venezuela. These results reveal the importance of characterizing tropical populations of *Coccidioides* as they harbor distinct genetic variants, and likely phenotypic differences, among populations. These populations can act as donors of variation and contribute to the evolution of other populations in subtropical and temperate regions.

## RESULTS

### SNPs.

Reads from the 51 *C. posadasii* previously sequenced genomes were aligned to the reference *C. posadasii* strain Nuevo Leon-1. This alignment had 261,105 single nucelotide polymorphisms (SNPs). When we included the *C. immitis* 202 strain as the outgroup, the alignment had 464,281 SNPs. The difference in the numbers of polymorphic sites between these two alignments reflects the genetic differentiation between *C. immitis* and *C. posadasii*.

### Phylogenetic analyses and time to most recent common ancestor.

First, we found that the general time reversible (GTR) model coupled with empirical base frequencies (+F), ascertainment bias correction (+ASC), and nine categories of FreeRate model (+R9) was the best fit for the *C. posadasii* strain Nuevo Leon-1 alignment. The transversion model (TVM) coupled with ascertainment bias correction (+ASC) and four categories of discrete gamma model (+G4) was the best fit for the alignment for the *C. posadasii* and *C. immitis* 202 alignment. We then used these alignments and models of molecular evolution to generate a maximum likelihood phylogenetic tree. We rooted the tree with *C. immitis*, the closest relative of *C. posadasii*.

**FIG 1:**
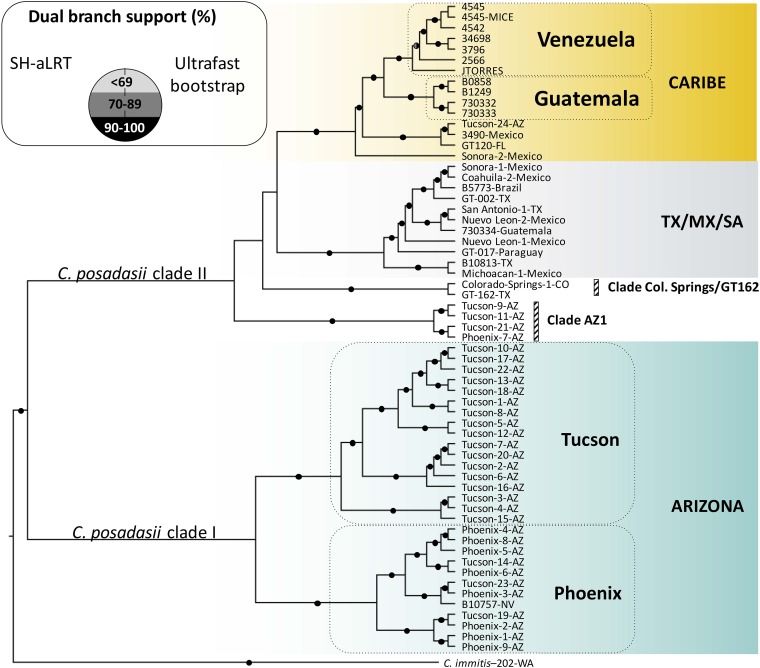
Maximum likelihood (ML) phylogenomic analyses among members of the *C. posadasii* complex. The ML tree was rooted with the *C. immitis* strain 202, and clades are displayed proportionally to the branch length of the clades since the majority of SNPs are derived from the overall *C. posadasii*/*C. immitis* divergence. Dual branch support was evaluated using 1,000 ultrafast bootstraps coupled with a Shimodaira-Hasegawa-like approximate likelihood ratio test and are displayed next to the clades. The approximate location of collection of each strain was added next to the taxon that represents the country (full names) or the American states as follows: Arizona (AZ); Nevada (NV), Texas (TX), Colorado (CO), Washington (WA), and Florida (FL).

The resulting maximum likelihood tree revealed that *C. posadasii* encompasses two major clades, one formed exclusively by Arizona isolates (*C. posadasii* clade I) and the other more heterogeneous clade that includes all the isolates from South America, Central America, and Texas, as well as a few isolates from Arizona (*C. posadasii* clade II [Fig. 1]). The branches shown in Fig. 1 have bootstrap and SH-aLRT (Shimodaira-Hasegawa-like approximate likelihood ratio test) support of over 90%. As expected, the longest branch in the tree (i.e., genetic distance) is the branch that separates *C. posadasii* and *C. immitis*, the two species of *Coccidioides*. The 95% highest posterior densities (HPDs) showed that the conservative age root to the whole *Coccidioides* tree (spanning both 5.1 and 12.8-million years ago [mya] root age hypotheses) is within 4 to 15 mya. Most population splits happened within ∼150,000 to 250,000 ya. The times to most recent common ancestors (TMRCAs) of Venezuelan samples were inferred to be within ∼1,400 to 5,500 ya (birth-death tree prior), or 1,500 to 5,700 ya (Bayesian skyline tree prior); The TMRCA of Guatemala samples was within ∼22,000 to 87,000 ya (birth-death), or 22,500 to 87,600 ya (Bayesian skyline) (see Table S2 in the supplemental material). A precise determination of divergence of populations is complicated, and TMRCAs in these analyses should be considered estimates (33).

The *C. posadasii* clade I contains two main groups and harbors the majority of the Arizona strains; one composed of isolates from Tucson/Pima County, and one mostly (but not exclusively) composed of isolates from Phoenix/Maricopa County (Fig. 1). These localities are both in the state of Arizona, USA. This level of microgeographic structure is puzzling because of the geographical proximity between the two sites (160 km) and because the majority of these isolates were collected from human patients. One would expect people to move freely between these two localities. This pattern might suggest that even though people can move between these two cities, there is a high level of predictability as to where infections were contracted (i.e., the patient’s main residence).

The *C. posadasii* clade II encompasses a more diverse geographical sample that includes isolates from North, Central, and South America (Fig. 1). This clade is composed of four subgroups. The first one is the Caribe clade, which is formed by isolates mostly collected in localities surrounding the Caribbean Sea, including isolates from Venezuela, Guatemala, and Florida. This is one of the longest branches within *C. posadasii*. Venezuela forms a monophyletic group which is sister to Guatemala. In effect, the Caribbean group is paraphyletic when Venezuela is not included in the topology (data not shown). The second group of the non-Arizonan isolates of *C. posadasii*, encompassing isolates from South America, appear nested within isolates from Texas and Mexico (henceforth referred to as TX/MX/SA [Fig. 1]). The existence of this group, which harbors South America and North American isolates, is consistent with (but does not uniquely support) the hypothesis that a genetically diverse group from North America underwent a population bottleneck while expanding south, and gave rise to genetically depauperate populations in South America (28). Interestingly, an isolate from a Guatemalan patient does group with TX/MX/SA; however, as noted in Engelthaler et al., this isolate was from a patient with travel history to Texas (21). A third group is formed by 4 isolates from Arizona (Clade AZ1 in Fig. 1). Even though these clinical isolates were collected in the same localities as the isolates from the Arizona population, they are not associated with *C. posadasii* clade I (Arizona [Fig. 1]). This suggests that these infections might have been acquired in a location different from southern Arizona (Tucson or Phoenix) or that additional phylogenetic clades remain to be defined with additional sampling. Finally, we find a small phylogenetic group composed of one isolate from Texas and one from Colorado (Fig. 1). A more systematic sampling is needed in areas of endemicity of Latin America to fully understand the genealogical and geographic relationships among *C. posadasii* groups, but the results from this genome-wide phylogenetic tree suggest there is differentiation among *C. posadasii* populations based primarily on geographic origin.

### Population structure.

**FIG 2:**
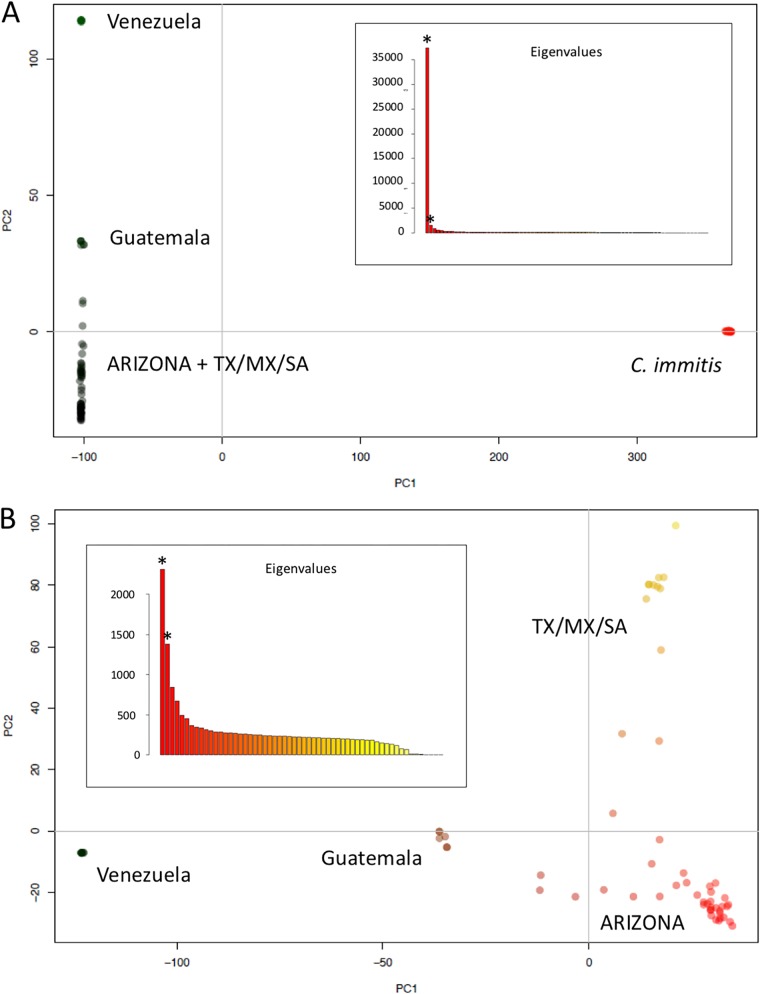
Principal-component analysis (PCA) reveals population structure of *C. posadasii*. (A) PCA, including *C. immitis* and *C. posadasii*. PC1 (75.34%) separates species of *Coccidioides*, while PC2 (3.03%) suggests the existence of cryptic populations within *C. posadasii*. (B) PCA, including only isolates from *C. posadasii*. PC1 (14.26%) coordinates separates isolates from Venezuela and isolates from the rest of the geographic range. PC2 (8.52%) discriminates the TX/MX/SA and Arizona populations. The insets on each panel show the eigenvalues that were used to plot the two principal coordinates. (Asterisks represent the first 2 PCs.)

We next explored the partitioning of genetic diversity within *C. posadasii* using population genetics approaches. First, we assessed how genetic diversity was apportioned among isolates with a principle-component analysis (PCA). We performed the analysis in two different ways. First, we included *C. immitis* and *C. posadasii* in the sample (Fig. 2A). As expected, principal component 1 (PC1 [75.34% of the variance]) separates the two *Coccidioides* species. Consistent with previous findings, we see no intermediate isolates, suggesting that even though admixture between the two species of *Coccidioides* has occurred (27, 34), the amount of genetic exchange between species is small enough to not affect species delineations. Notably, PC2 (3.03% of the variance) differentiates between the Arizona plus TX/MX/SA and Venezuela populations, with the Caribbean (e.g., Guatemala, and Florida) group appearing as intermediaries between these two populations. PC2 thus corresponds to the population variation within *C. posadasii*. Interestingly with this analysis, the Guatemala population does not appear as a separate lineage but instead appears to be an intermediate between the Arizona and Venezuela populations.

The PCA that only included *C. posadasii* showed similar patterns but added more resolution to the differentiation within *C. posadasii* (Fig. 2B). PC1 (14.26% of the variance) separates Venezuela and the remainder of the *C. posadasii* populations. PC2 (8.52% of the variance) separates the TX/MX/SA population from the rest of the *C. posadasii* Arizona populations. We find isolates that show genetic variation patterns that are intermediate between populations. The isolates from Guatemala and the isolates GT-120 (Miami, FL), Tucson 7, and Tucson 2, appear as intermediaries between the Arizona and Venezuela clusters (Fig. 2B; PC1). This result is consistent with the PCA that included both species of *Coccidioides* in the previous analysis. Three isolates (Sonora 2, Michoacán 1, and B01813-TX) from Mexico and Texas appear as intermediates between Arizona and TX/MX/SA (Fig. 2B; PC2). These results are suggestive of population differentiation among populations between different geographical regions of *C. posadasii* and a degree of genetic exchange between these populations.

**FIG 3:**
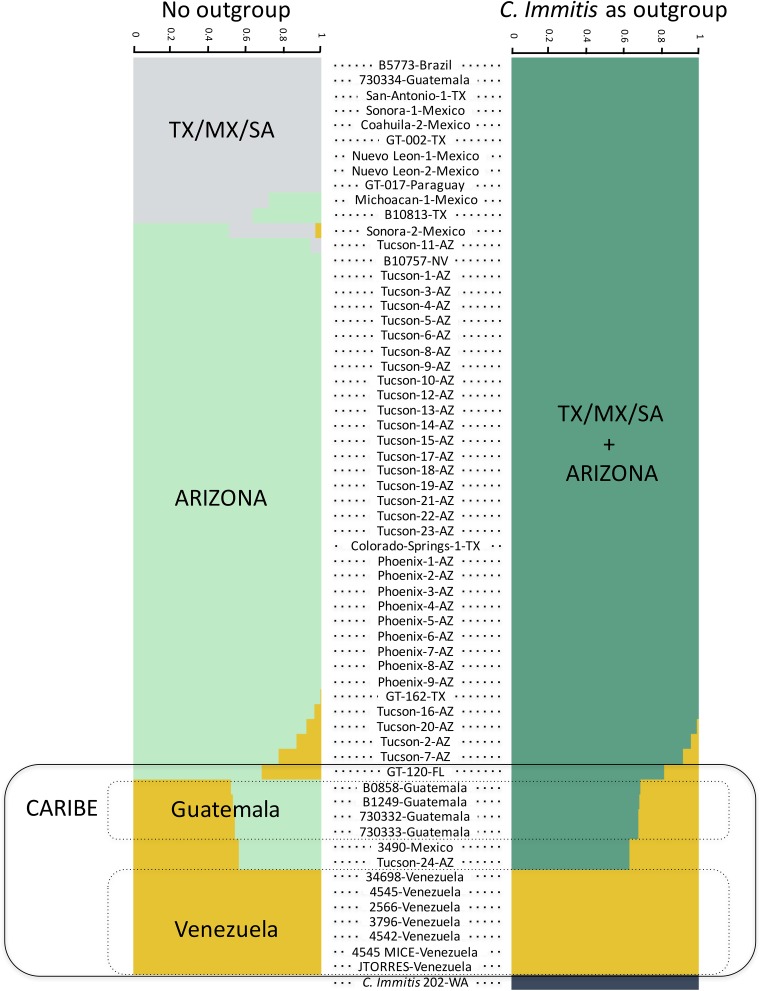
Strong population structure within *C. posadasii*. Population structure plots based on Bayesian posterior probabilities implemented in fastSTRUCTURE. We did the analysis in two ways, first using *C. immitis* as an outgroup for *C. posadasii* or using only *C. posadasii* strains. Each row represents an individual. The heights and colors of percentage of each population represent the probability of belonging to a given cluster.

Second, we used fastSTRUCTURE with the admixture mode to infer the most likely number of groups in *C. posadasii*. When we include *C. immitis*, the method infers three clusters: *C. immitis*, *C. posadasii* from Arizona plus TX/MX/SA, and *C. posadasii* from Venezuela (Fig. 3). This result is qualitatively similar to the result from the PCA using the same data set. When the analysis is run without *C. immitis*, we find a similar result. The genomic data reveal the existence of a broader Venezuela group containing isolates from the Caribbean region, an Arizona group, and a third group formed by the TX/MX/SA isolates. The detection of the latter cluster is the main difference from the analysis run with *C. immitis*. These two results are consistent with the results from the two PCAs (Fig. 2) and suggest the existence of strong population stratification within *C. posadasii*. Notably, fastSTRUCTURE infers that the isolates from the Caribbean/Guatemalan group are intermediates between Arizona and Venezuela in the PCA and are not as clearly assigned to a cluster. These show a high proportion of ancestry associated with the Venezuela group (*P* ≥ 0.85), but there is also a nontrivial proportion of ancestry that is associated with the Arizona group (*P* ≈ 0.15). The results are also largely consistent with but not identical to those from previous attempts to determine the partition of genetic variation within *C. posadasii* (21); namely, we find that the Caribbean group is not an isolated population but instead is a population that cannot be assigned to either Venezuela or North America. The reasons for this conflict are explored below.

### Genetic distances.

Next, we looked for evidence of cryptic speciation within *C. posadasii*. We measured the mean genetic distance between individual genomes in all pairwise comparisons to get a proxy of within-population diversity and potential between population differentiation. Consistent with previous reports, *C. immitis* and *C. posadasii* are highly differentiated; the magnitude of interspecific differentiation is over 10 times larger than within-species variation. The genetic distance value among populations is around 6% in all pairwise comparisons. Within each population, most π values are close to 5% (i.e., π_Tucson_ = 5.7%, π_Phoenix_ = 5.3%, and π_TX/MX/SA_ = 4.5%). This value is in line with the levels of intraspecific variation described for other fungal species (35). However, the Caribbean group shows π values that are much lower. Diversity in Guatemala is three times lower than in North American groups (π_Guatemala_ = 1.5%). A more extreme example is that of Venezuela, where diversity is ∼50 times lower than that of other *C. posadasii* populations (π_Venezuela_ = 0.09%). We next compared these values to determine whether interpopulation pairs show a higher genetic distance than intrapopulation pairs. We find that this is the case in most comparisons (see Table S3 in the supplemental material, which shows results from asymptotic two-sample permutation tests). These differences are consistent across groups, and the magnitude of divergence among populations is slightly higher than that within populations but does not clearly indicate the existence of cryptic species within the Caribbean group.

### Mating type and recombination analyses.

One possibility that might explain the low heterozygosity in the Venezuela population might be complete asexual reproduction due to the absence of one of the mating types, thus reducing recombination rates. All 7 isolates from the Venezuelan phylogenetic group harbor the *MAT1-2* idiomorph in their haploid genomes, while all other *C. posadasii* populations have equal distribution of *MAT1-1* and *MAT1-2* idiomorphs (21, 36). This prevalence of a single mating type is similar to observations for the *C. immitis* Washington population (21), which suggests that population bottlenecks associated with range expansion might result in a single mating type and a clonal population structure. Phylogenetic network and recombination analysis within different *C. posadasii* populations together indicate that Venezuela is a clonal lineage (*P* = 1, with no conflicting edges), because no incongruent signals in the SNP matrix were found in the neighbor-net network and pairwise homoplasy index (PHI) test analyses. On the other hand, the Arizona population showed a strong signal of recombination (*P* = 0.0, presence of conflicting edges), which supports the balanced *MAT1-1* and *MAT1-2* distribution observed being consistent with outcrossing and sexual reproduction (see Fig. S1 in the supplemental material).

10.1128/mBio.01976-19.1 FIG S1 Phylogenetic network and recombination analysis within different *C. posadasii* populations. Panel A shows the neighbor-net network across all *C. posadasii* genomes analyzed and highlights the individual tested populations. Intrapopulation neighbor-net networks were also produced for *C. posadasii* Arizona (B), TX/MX/SA (C), AZ clade 1 (D), Venezuela (E), and Guatemala (F). Connected edges represents incongruent signal in the genomic data. Results for PHI tests are displayed above each individual phylogenetic network: *P* = 0.0 indicates recombination, while *P* = 1 indicates clonality. Download FIG S1, PDF file, 2.6 MB. Copyright © 2019 Teixeira et al. 2019 Teixeira et al. This content is distributed under the terms of the Creative Commons Attribution 4.0 International license.

### Admixture.

**FIG 4:**
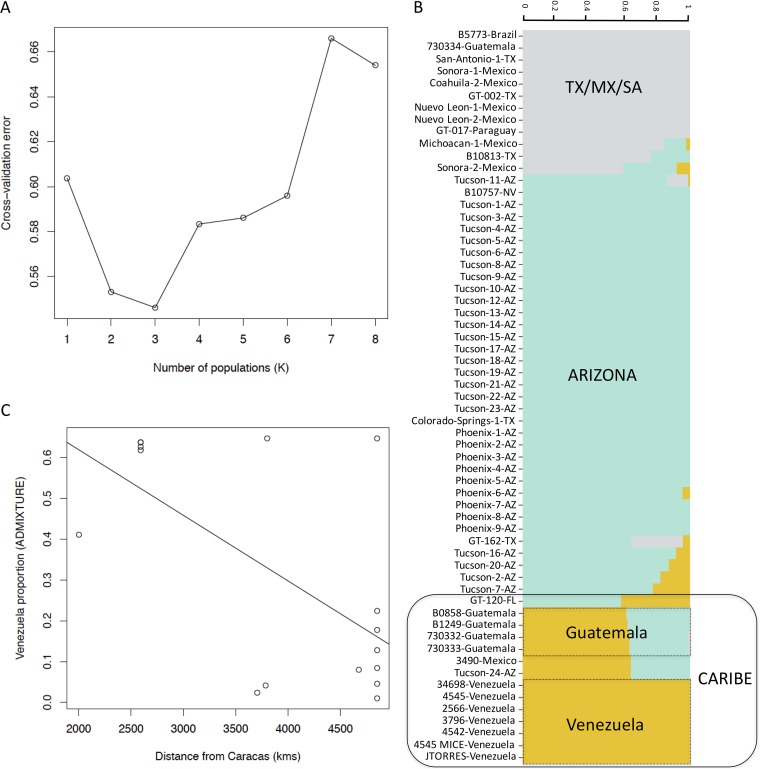
Within-species admixture in *C. posadasii*. (A) Cross-validation error learning curve of the log likelihood values collected by ADMIXTURE from *K* = 1 to *K* = 8 population scenarios. (B) Individual admixture proportions of *C. posadasii* isolates as inferred with ADMIXTURE. The height and colors of percentage of each population represent the probability of a given strain belonging to a given population. (C) The proportion of the Venezuela admixture decreases as the distance from Caracas increases.

PCA and fastSTRUCTURE results suggest that some isolates have mixed genetic ancestry between divergent *C. posadasii* populations. We studied the geographical partition of shared ancestry in the Caribbean group within *C. posadasii*. We used ADMIXTURE to infer the contribution from the minor population (i.e., Venezuela) as a proxy for the proportion of admixture in each of the Caribbean group isolates. The results from ADMIXTURE show a similar clustering to the one revealed from fastSTRUCTURE and also suggest the presence of three populations within *C. posadasii* (Fig. 4A). The contributions of each of the three resulting populations to the admixed individuals of *C. posadasii* are shown in Fig. 4B. Next we studied the broad distribution of Venezuela ancestry across geography. Using the inferred proportion of Venezuela ancestry, we tested whether there was a relationship between the proportion of Venezuela contribution and the distance to the center of the geographical distribution of the parental population. Within the Caribbean population, we find that the proportion of Venezuela ancestry decreases slightly but not significantly as the distance from Caracas increases (ρ_Spearman_ = −0.3562, *P* = 0.088 [Fig. 4C]). These results suggest that the Caribbean populations are akin to a contact zone between the Arizona and Venezuela populations. More generally, the results collectively show that in spite of the strong differentiation between *C. posadasii* populations there is evidence of admixture and gene exchange.

## DISCUSSION

In this study, we assess the magnitude of the differentiation within *C. posadasii* using whole-genome sequence comparisons. The magnitude and partitioning of genetic variation in human fungal pathogens are a key, yet underappreciated, aspect of pathogen biology. *Coccidioides* represents a clear example of this paucity. Even though coccidioidomycosis exists in Central and South America, very little is known about the biology of the fungus in these regions. Preliminary population assessments indicated that isolates from Central America belong to *C. posadasii*, but these are genetically differentiated from both the *C. posadasii* TX/MX/SA clade and the Arizona clade (22). Notably, little is known in terms of the genetic diversity of isolates from xeric environments from South America, which poses the possibility that our current understanding of the relationships between *C. posadasii* isolates is incomplete.

Epidemiological studies show that coccidioidomycosis in South America is mostly caused by *C. posadasii* (19, 28, 37). Molecular analyses of soil DNA revealed that *Coccidioides* is common in xeric environments of Venezuela: all sampled sites (*n* = 15) were positive for *Coccidioides*, and sequencing of one of the ribosomal internal transcribed spacers (ITS2) suggested that all the environmental samples from the region are, indeed, *C. posadasii* (32). Nonetheless, the genotypes were diverse and encompassed multiple ITS2 haplotypes. If *C. posadasii* is so common in the soil in Venezuela, why is coccidioidomycosis so rare in the same area? One possibility is that the *C. posadasii* Venezuela lineage is less infective than other *Coccidioides* lineages. Since *C. posadasii* in Venezuela seems to have undergone a strong bottleneck (28), it is possible that the fungus has accumulated deleterious mutations that diminish its ability to establish infection in humans or other hosts. Another possibility is that the Venezuela lineage is less virulent than other lineages. In this scenario, even if the fungus comes into contact with humans and has the same ability to cause infection as other lineages, the disease is more likely to be asymptomatic. This would be consistent with the fact that a large proportion of Venezuelans have a positive reaction to coccidioidin intradermic tests. The proportion of reactors in Venezuela is comparable to other human populations in regions of endemicity (38, 39). Variation in the ability to cause disease, either because of genotypic differences in the ability to infect or to induce disease, has been reported for other fungi of the Onygenales. Species within the *Histoplasma* genus, for example, show differences in fungal burden, disease kinetics, symptomology, and cytokine responses in controlled infections (40). Currently there is no definitive evidence of these phenotypic differences among lineages of *Coccidioides*. It is also possible that coccidioidomycosis is underreported in Venezuela compared to other regions of endemicity. Additionally, arid regions in South America tend to be associated with lower population densities and lower socioeconomic status than coastal cities. Certainly, all these possibilities are not mutually exclusive. Defining the relative importance of these factors that may contribute to the low numbers of coccidioidomycosis in South America will required a combination of population genetics, functional studies, and fine-scale and comparable epidemiological studies paired with environmental surveys in multiple *Coccidioides* populations from different regions of endemicity.

One proposed hypothesis to explain *C. posadasii* genotypic distributions in South America is dispersion associated with human migration from North America (28). Early settlements of humans in Venezuela (Taima-Taima archeological site, Falcon State) were dated to 15 kya (41). Most population bifurcations happened no less than 152,000 ya (Table S2). Phoenix/Tucson, the most recent divergence, took place at least 66,000 ya. The Venezuelan population originated at least at 148,000 ya, suggesting its origin happened before the oldest human occupation in the Americas known to date (42). The confidence intervals of these dates do not overlap the more well-accepted understanding of human colonization of the Americas (less than 17,000 years ago [43–45; but see reference 46]). In fact, there are several alternative hypotheses. One possibility is that the mutation rate, which was used to convert genetic differentiation to years of divergence, differs across multiple groups of *Coccidioides*. However, the difference in mutation rates between *Coccidioides* lineages would have to high (∼10×) to qualitatively change our conclusions. Second, it is possible the Venezuela lineage originated somewhere else (i.e., North America) and went extinct in its location of origin. If this lineage was only able to establish itself in Venezuela, then the hypothesis of human-driven migration of *C. posadasii* to South America would be compatible with our results. Third, other animal migrations and host-pathogen associations have not been explored. The “Great American Biotic Interchange” resulted in many species moving from North America to South America and vice versa, and any of these could be preferred or novel host species. Finally, the vast majority of isolates analyzed to date are derived from human patients, and we cannot be fully confident of their site of origin or that human clinical isolates represent overall genetic diversity of *Coccidioides*. The patterns of dispersion of fungal pathogens, including *C. posadasii*, remain an underexplored topic.

The study of population structure in fungi has been almost exclusively applied to defining cryptic species. These lineages, which show the genetic signature of reproductive isolation (i.e., the phylogenetic species concept, reviewed in reference 47), are morphologically similar and were not known to be distinct before DNA sequencing became possible (reviewed in reference 48). This approach has led to the conclusion that the number of species of fungi is significantly underestimated, because each species recognized by morphology harbors several phylogenetic species (4). Delineating species boundaries has important consequences for epidemiology and our understanding of pathogenesis; human pathogens previously assigned to a single species have been found to show different levels of virulence and in some cases cause different diseases. The case of *Histoplasma* exemplifies this utility. Recently, four species of *Histoplasma* were formally adopted following genomic differentiation. These lineages differ in their genome sizes, in their abilities to cause disease, and their geographical distributions (49). Analyses of gene flow show that these species exchange genes rarely but that most introgressions are found at low allele frequency, which in turn might indicate the possibility that these exchanged alleles are deleterious or slightly deleterious.

The magnitude of genetic differentiation seems to be insufficient to describe the different lineages of *C. posadasii* as well-formed different species. The case of the Venezuela clade in which the magnitude of interpopulation genetic distance is higher than the variation within Venezuela is worth highlighting. This pattern is caused by two main factors. First, the Venezuela lineage shows a reduction in genetic diversity of 50× compared to other *C. posadasii* populations. Yet, the magnitude of interpopulation differentiation between Venezuela and other populations is similar to the magnitude of variation within the other populations (Table S3). The results suggest that there is strong population structure within species of *Coccidioides* which might be a signal of incipient and recent speciation. Nonetheless, differentiation between incipient speciation and strong population structure is a major challenge (50, 51), which cannot be tackled without *prima facie* evidence of reproductive isolation, and to date, no sexual cycle has been defined for *Coccidioides*. Additionally, the lower variability in Venezuela leads to a skewed ratio of inter- to intrapopulation variation in pairwise comparisons involving Venezuela and, to some extent, Guatemala, but this is not caused by an increase in the interpopulation differentiation but by an extreme reduction in intraspecific variation. Moreover, we observe a single mating type (*MAT1-2*) within the Venezuelan population and according to the typical bipolar mating system of Eurotiomycetes, both opposite mating type cells (*MAT1-1* and *MAT1-2*) are need for sexual recombination. Thus, we conclude that this is likely an extreme bottleneck event and not a signature of speciation.

Our analyses are parallel approaches used to quantify interspecific gene flow in pathogenic fungi, but our focus differs from these studies: our goal was to determine if populations of *C. posadasii* are differentiated and exchange alleles. Understanding geographic population subdivision is a prerequisite to address if genetic variation associated with virulence might be originating across the geographic range of a species. Intraspecific population structure and speciation are part of a continuum that is modulated by the strength of reproductive isolation and the extent of genetic divergence between lineages (52, 53). The continuous nature of divergence and speciation makes the distinction of species boundaries challenging because population genetics estimates of differentiation cannot distinguish between nascent species and well-structured and old populations of the same species (51, 54). This is especially true for populations that occur in allopatry and do not interbreed.

Species that have achieved genome-wide reciprocal monophyly will show fixed differences, which allows for detection of alleles that have crossed species boundaries after hybridization (e.g., 27 and 55). Populations from the same species, or even incipient species, are less likely to show these fixed differences, and analyses of introgression must be done based on differences of allele frequencies. This approach has proven useful in human populations (56) and maize (57) (reviewed in reference 58). In the case of *C. posadasii* populations, it is possible that shared ancestry among populations is not caused by gene exchange but by incomplete lineage sorting (ILS) across different populations. This retention of ancestral alleles might be due to balancing selection, or simply by chance (59, 60). As genetic divergence accumulates, the likelihood of retaining ancestral polymorphism by chance decreases; thus, when divergence is still recent (populations or incipient species) ILS should be common (59, 60).

The *Coccidioides posadasii* population from Venezuela is not the only fungal pathogen from this country that shows signatures of genetic isolation. For the Onygenalean fungal pathogen in the family Ajellomycetaceae *Paracoccidioides* spp., sampling efforts have been roughly equivalent in multiple countries. In the specific case of *Paracoccidioides*, two species coexist in the same geographic range: one endemic species, Paracoccidioides venezuelensis, and a species with broader distribution, Paracoccidioides americana, found in Venezuela and Brazil (61, 62). Whether the environments of Venezuela facilitate divergence or whether this is a case of differences in sampling effort remains unknown. These two alternatives are both possible, as xeric environments in South America have not been systematically sampled for *Coccidioides*, which is in the family Onygenaceae, and it is possible that each xeric environment within South America harbors its own lineage of *Coccidioides*.

Hybridization and gene exchange seem to be a common occurrence during the evolutionary history of fungal pathogens. For example, *Paracoccidioides* species show complete mitochondrial capture which is reflected in the discordance between nuclear and mitochondrial gene genealogies (63). Histoplasma ohiense and Histoplasma mississippiense show evidence of admixture in their nuclear genome but such exchanged alleles are at low frequency (49, 55). *Cryptococcus* and *Candida* species also show evidence of shared genetic variation (64, 65). This pattern is not limited to human pathogens. Plant pathogens and saprobic fungi also show evidence of gene exchange but the features that govern the magnitude of gene exchange remain unknown (e.g., 66, 67; reviewed in reference 68). The general patterns of gene flow are consistent with trends observed in other taxa, but the underlying mechanisms still need to be formally tested in fungi.

Genetic variation among all taxa is determined by the generation of new variants by mutation, migration, and recombination (35, 69). Geography plays a fundamental role in maintaining variants over time (70). Semi-isolated lineages might serve as reservoirs of variation which might feed into the main gene pool of a species by sporadic gene flow. These metapopulation dynamics in which populations are connected to each other, and there is the possibility for population divergence and contact, is not exclusive to pathogens. In the case of *Coccidioides*, a systematic sampling of both host and environmental isolates in suitable environments across the entire geographic range is necessary to assess how selection might influence the persistence of new mutations, and the evolutionary history of the species. More generally, approaches that incorporate spatial and temporal partitioning of genetic variation in pathogens will be crucial to understanding the factors that shape the genome and species history of organisms crucial to human well-being.

## MATERIALS AND METHODS

### Fungal strains, DNA extraction, and DNA sequencing. (i) Coccicioides isolates.

*Coccidioides* isolates were retrieved from human clinical specimens collected at the at the Servicio Autónomo Instituto de Biomedicina Dr. Jacinto Convit, Caracas, Venezuela (6), the Valley Fever Center for Excellence, Tucson, AZ (1), or the UC Davis Center for Valley Fever, UC Davis Health, Davis, CA (3). Clinical specimens were initially isolated on Mycosel agar (BD Biosciences) or Sabouraud agar with 100 μg/ml chloramphenicol and cycloheximide, and fungal isolates were subjected to serial passage onto 2× GYE medium (1% [wt/vol] Difco yeast extract and 2% [wt/vol] glucose) for fungal colony stabilization and to detect any potential contamination. *Coccidioides* arthroconidia were harvested, preserved in 25% glycerol, 0.5% glucose, and 0.25% yeast extract storage medium, and finally stored at –80°C.

**(ii) DNA extraction.** To culture isolates and extract DNA, we grew *Coccidioides* (isolates listed in Table S1 in the supplemental material) under biosafety level 3 (BSL3) conditions either at the Pathogen and Microbiome Institute, University of Northern Arizona, Flagstaff, AZ, or at the Servicio Autónomo Instituto de Biomedicina Dr. Jacinto Convit, Caracas, Venezuela. We started all fungal cultures from previously stored single-spore isolate glycerol stocks kept at –80°C on 2× GYE medium for mycelial propagation at 28°C for 14 days. We harvested ∼500 mg of mycelia using a cell scraper (VWR, Radnor, PA), and this material was used as input for total DNA extraction using the UltraClean microbial DNA isolation kit (Qiagen) according to the manufacturer’s protocol. We then confirmed the sterility of all DNA before removing the DNA preparations from the BSL3 facility for further sequencing by plating 5% of the eluted material onto 2× GYE medium, and growth was evaluated after 72 h at 28°C. Once removed from the BSL3 facility, we estimated DNA purity and concentration using spectrophotometry on the NanoDrop ND-1000 system (Thermo Fisher Scientific).

10.1128/mBio.01976-19.2 TABLE S1 SRA accession number, isolate identifier, species, publication source, and origin of isolation for each *Coccidioides* strain used for phylogenomics and population genetic analyses. Download Table S1, XLSX file, 0.01 MB. Copyright © 2019 Teixeira et al. 2019 Teixeira et al. This content is distributed under the terms of the Creative Commons Attribution 4.0 International license.

10.1128/mBio.01976-19.3 TABLE S2 The 95% HPDs showed that the root of the whole tree is at least 4,000,000 ya, with most bifurcations happening no less than 152,000 ya. Phoenix/Tucson, the most recent divergence, took place at least 66,000 ya. The Venezuelan population originated at least at 148,000 ya, suggesting the population originated before the oldest human occupation in the Americas. Download Table S2, XLS file, 0.03 MB. Copyright © 2019 Teixeira et al. 2019 Teixeira et al. This content is distributed under the terms of the Creative Commons Attribution 4.0 International license.

10.1128/mBio.01976-19.4 TABLE S3 Genetic distances between all pairwise population comparisons within *C. posadasii*. We used asymptotic two-sample permutation tests to compare intra- and interspecific differences. Download Table S3, DOCX file, 0.01 MB. Copyright © 2019 Teixeira et al. 2019 Teixeira et al. This content is distributed under the terms of the Creative Commons Attribution 4.0 International license.

**(iii) Library preparation and sequencing.** We prepared sequencing libraries for the 10 *de novo* sequenced isolates using the Kapa Biosystems kit (Kapa Biosystems, Woburn, MA) and ∼1 μg of DNA of each isolate according to the manufacturer’s protocols. We then multiplexed individual libraries using 8-bp indexes and quantified using quantitative PCR (qPCR) in a 7900HT system (Life Technologies Corporation, Carlsbad, CA) using the Kapa library quantification kit (Kapa Biosystems, Woburn, MA). We pooled libraries that were quantified using qPCR as described above and sequenced them on an Illumina HiSeq 2500 instrument (Illumina, San Diego, CA) at the Translational Genomics Research Institute (TGen), aiming for a coverage of 100× per isolate. Resulting paired-end reads had a length of 125 bp.

### Public data.

We obtained 72 publicly available sequence reads for *C. posadasii* (51 reads) and *C. immitis* (21 reads) isolates from the Sequence Read Archive (SRA). All accession numbers are listed Table S1.

### Read mapping and variant calling.

First, we removed Illumina adaptors using Trimmomatic v0.36 from the reads obtained for the 10 sequenced isolates (71). To improve *de novo* references, we used the Unmanned Genome Assembly Pipeline (UGAP [https://github.com/jasonsahl/UGAP]), which uses the genome assembly algorithm SPAdes v3.10.1 (72) as well the Pilon toolkit v1.22 (73). We obtained publicly available *Coccidioides* raw reads from the Sequence Read Archive (SRA) deposited under accession no. SRR3468064 (*C. posadasii* Nuevo Leon-1) and SRR1292225 (*C. immitis* strain 202). We used Burrows-Wheeler Aligner (BWA) v0.7.7 (74) to align reads to each of the assembled references: *C. posadasii* strain Nuevo Leon-1 (21) or *C. immitis* strain 202 (75). We removed mismatching intervals with the RealignerTargetCreator and IndelRealigner tools available in the GATK toolkit v3.3-0 (76, 77). To call SNPs, we used UnifiedGenotyper, setting the parameter “het” to 0.01. Finally, we filtered the .vcf files using the following parameters: QD = 2.0 ǁ FS_filter = 60.0 ǁ MQ_filter = 30.0 ǁ MQ_Rank_Sum_filter = −12.5 ǁ Read_Pos_Rank_Sum_filter = −8. SNPs with less than 10× coverage or with less than 90% variant allele calls or that were identified by NUCmer (78) as being within duplicated regions in the reference were removed from the final data set. In total, our data set was composed of 82 *Coccidioides* genomes.

### Phylogenetic tree.

To study the genealogical relationships among *C. posadasii* isolates, we built a maximum likelihood (ML) phylogenetic tree using genome-wide SNP data. We used concatenated genome-wide SNPs as *Coccidioides* as we expect low levels of genealogical discordance (79, 80). We first obtained whole supercontig sequences for each individual from the variant call format (VCF) file using the FastaAlternateReferenceMaker tool in GATK. Next, we used IQ-TREE to infer the most likely tree with maximum likelihood (81); we used the -m TEST option (jModelTest [82]) for automatic molecular evolution model selection. We calculated the support of each branch on the resulting topology, using 1,000 ultrafast bootstraps coupled with a Shimodaira-Hasegawa-like approximate likelihood ratio test (SH-aLRT) (83, 84). Tree topologies were visualized using FigTree v1.4.2 (http://tree.bio.ed.ac.uk/software/figtree/).

### Approximate time to most recent common ancestor.

We used BEAST2 (v2.6.1 [85]) to infer molecular dating estimates. We used the SNAPP module, which under a coalescent framework estimates concomitantly the joint effective population size, molecular rates, and divergence times (86). We followed the approach used by Stange et al. (87) in which a molecular clock (the available clock model currently supported in SNAPP) and effective population sizes are shared between all species, which according to their analyses led to accurate dating estimates. We assumed the age of the root to be within a normal distribution of mean = 5.1 (97) and standard deviation being 10% of that variation; we then extrapolated the 95% highest posterior density (HPD) obtained, to match a root age prior (mean, 12.8; standard deviation [SD], 1.28), using 12.8 mya as the TMRCA based on a study by Fisher et al. (98). After this first round of analyses, we selected only the samples from Venezuela and Guatemala for a new set of BEAST2 runs, to infer the TMRCA of those populations. We set the root age prior for this new run as a normal distribution, with its 95% prior density falling within the 95% HPD bounds obtained in the previous step; we also set the clock rate prior as a normal distribution following the same approach (i.e., extrapolation from the runs, including the whole data set of the first round of analyses). For the second round of analyses, we used two different tree priors on the analysis of the concatenated SNP data—a birth-death prior and an extended Bayesian skyline prior—to be more conservative in our estimation. An extrapolation to a normal distribution (12.8, 1.28) was also obtained, which was based on the root age of 12.8 mya (98). For each scenario, we ran two Markov chain Monte Carlo (MCMC) runs until convergence was detected and effective sample sizes were 200, as revealed in Tracer v1.7 (63). We then summarized the two runs for each case using treeannotator (within the Beast distribution) after discarding the burn-in, summarizing 95% HPD of divergence times.

### Population structure.

We studied the population structure of *C. posadasii* using two different methods. First, we used principal-component analysis (PCA), which provides a graphical representation of the partition of genetic variance in a population sample. To do so, we restricted our data set to biallelic sites and used the R package *adegenet* (88). We used the function fasta2genlight to extract biallelic SNPs from VCF files (described above) and the function glPca to compute the first two principal components (PCs). We also report the percentage of genetic variance explained by each PC. Next, we inferred the most likely population clustering within *C. posadasii* using fastSTRUCTURE v1.0 (89). This tool calculates the most likely number of populations (*K*) and the probability that each strain belongs to each population. SNPs were assumed to be unlinked under the admixture model based on previous assessments of linkage disequilibrium in *Coccidioides* (90, 91), and data were transformed from haploid to diploid (creating all homozygous individuals), and the ancestry of each individual and correlated allele frequencies were simulated for a range of 2 to 8 populations. To infer the most likely true number of populations, we found the *K* with the lowest likelihood using the script chooseK.py (89), which parses the log files of each inferred value of *K* and infers the best-fitting model by maximizing the marginal likelihood.

### Genetic distances.

One of the proxies of cryptic speciation is that isolated species show considerably larger genetic differentiation than the magnitude of intrapopulation variation (80). We studied whether *C. posadasii* showed the signature of cryptic speciation. We measured the genetic differentiation between the nine groups defined by the phylogenetic analysis described above. To calculate the between group distance, we used *D_xy_*, the mean number of genetic differences between two genomes from different clusters. We compared the mean differentiation between two groups to the magnitude of genetic diversity within groups. To calculate the within group diversity, we used π, the mean number of genetic differences between two genomes from the same cluster. Genome-wide SNP data were loaded into MEGA7 (92), and the genetic groups were determined according to the groups revealed in the phylogenetic tree as follows: *C. immitis*, *C. posadasii* Venezuela, *C. posadasii* Guatemala, *C. posadasii* TX/MX/SA, *C. posadasii* clade AZ1, *C. posadasii* Phoenix, *C. posadasii* Tucson, and *C. posadasii* Tucson24/3490/GT120/Sonora. For pairwise comparisons, we use asymptotic two-sample permutation tests using the function *perm.test* (R library “exactRankTests”). Since there were two values of π for each value of *D_xy_*, we compared *D_xy_* values to the highest value of π in a pair of populations. We adjusted the significance threshold to account for multiple comparisons to *P* = 0.00138 (0.05/36).

### Mating type and recombination analysis.

We determined whether the genomes from the *C. posadasii* population from Venezuela have evidence of functional mating type loci based on sequence similarity to known mating type sequence. The Onygenalean mating type locus contains one of two forms of unrelated sequences (known as the idiomorph) in a syntenic but unique segment that gives mating type identity to fungal cells. To identify the mating type of the Venezuelan *C. posadasii* isolates, we followed the same strategy previously used to identify the *MAT1-1* or the *MAT1-2* locus in this pathogen (21, 36). Briefly, we used the full *MAT1-1* (containing the alpha-box *MAT1-1-1* gene [EF472259.1]) and *MAT1-2* (containing the HMG box *MAT1-2-1* gene [EF472258.1]) loci as reference sequences to query the Venezuelan *C. posadasii* read files. All isolates examined to date carry only one of these loci and are classified as either a *MAT1-1* or *MAT1-*2 genotype. Phylogenetic networks were produced using the neighbor-net network (NN) algorithm within each population to understand conflicting signal in genomic data. The overall recombination within each *C. posadasii* population was then analyzed using the PHI test, which also checks for incompatible nucleotide sites in a given DNA alignment. Both phylogenetic networks and PHI tests were performed on SplitsTree4 software (93).

### Admixture.

Previous studies have found that species of *Coccidioides* exchange alleles (27, 34). Our scope was different; we assessed whether populations within *C. posadasii* exchange genes. We estimated the proportion of admixture for each isolate using ADMIXTURE (94). We inferred the number of populations within *C. posadasii* by testing which *K* scenario had the lowest marginal likelihood, in a similar manner to that described for fastSTRUCTURE (described above). In the case of the Caribbean population, we regressed the proportion of admixture to the distance from the putative donor population, Venezuela.

Next, we used *f*_4_ statistics to assess the proportion of admixture. We restricted this analysis to the Guatemala population. We used admixtools (99) implemented in Treemix (95), and the option -k 1000 was used to group 1,000 SNPs to account for linkage disequilibrium. Assuming a given phylogeny, the *f*_4_ ratio allows estimation of the two mixing proportions during an admixture event, even without access to the precise populations that gave rise to the admixed lineage for the two ancestral populations. Since we have inferred the phylogenetic relationships among *Coccidioides* populations, we calculated the proportion of Venezuela ancestry in Guatemala following the equation proportion of admixture = *f*_4_(*AOXC*)/*f*_4_(*AOBC*), where *X* corresponds to the potentially admixed population (i.e., Guatemala). *O*, *B*, *A*, and *C* are four populations that are known to branch at four distinct positions along the phylogeny. *O* refers to the outgroup; in this case, we used *C. immitis*. *A* and *C* are the donors (or close relative to the donors) of the admixed lineage. In this case, we used *A* = Phoenix and *C* = Venezuela. *B* is a population that does not harbor introgression; we used *B* = Tucson. We found no difference in the estimations by swapping Tucson and Phoenix (data not shown).

Finally, we tested whether the contribution of Venezuela to the admixed population between Venezuela and Arizona followed the expectation that as distance from Venezuela increased, the contribution of the Venezuela population would decrease. Our hypothesis was that individuals from Central America were admixed and the product of gene exchange between the North American and South American *C. posadasii* populations. We used the proportion of admixture inferred from ADMIXTURE when *K* = 3 in *C. posadasii* (the best-fitting scenario [see Results]). We then calculated the distance between the collection site of a given sample (defined to the level of country or state) and Caracas using a haversine formula (96). The distance between Caracas and the closest major city to where the isolate was collected was calculated using the great-circle distance between two points (i.e., the shortest distance over the Earth’s surface). We used the approximate coordinates of seven locations to calculate the waypoints distance from Caracas (10.4806°N, 66.9036°W), because exact location of infection is unknown. We used the following sites and coordinates: Guatemala (15.7835°N, 90.2308°W), Coahuila (27.0587°N, 101.7068°W), Arizona (34.0489°N, 111.0937°W), Florida (27.6648°N, 81.5158°W), Texas (31.9686°N, 99.9018°W), Michoacán (19.5665°N, 101.7068°W), and Sonora (29.2972°N, 110.3309°W). We used a one-tailed Spearman correlation test using the R package “stats” (function “cor.test”).

### Data availability.

All the raw reads for the 10 isolates we sequenced in this article have been deposited at SRA under BioProject no. PRJNA438145 with sample accession no. SRR6830879 to SRR683088 (Table S1).
