# Supplementary figures and images for: Population Structure and Genetic Diversity among Isolates of Coccidioides posadasii in Venezuela and Surrounding Regions
Source: mBio. 2019 Nov 26;10(6):e01976-19. doi: 10.1128/mBio.01976-19 (PMC6879716; doi:10.1128/mBio.01976-19)

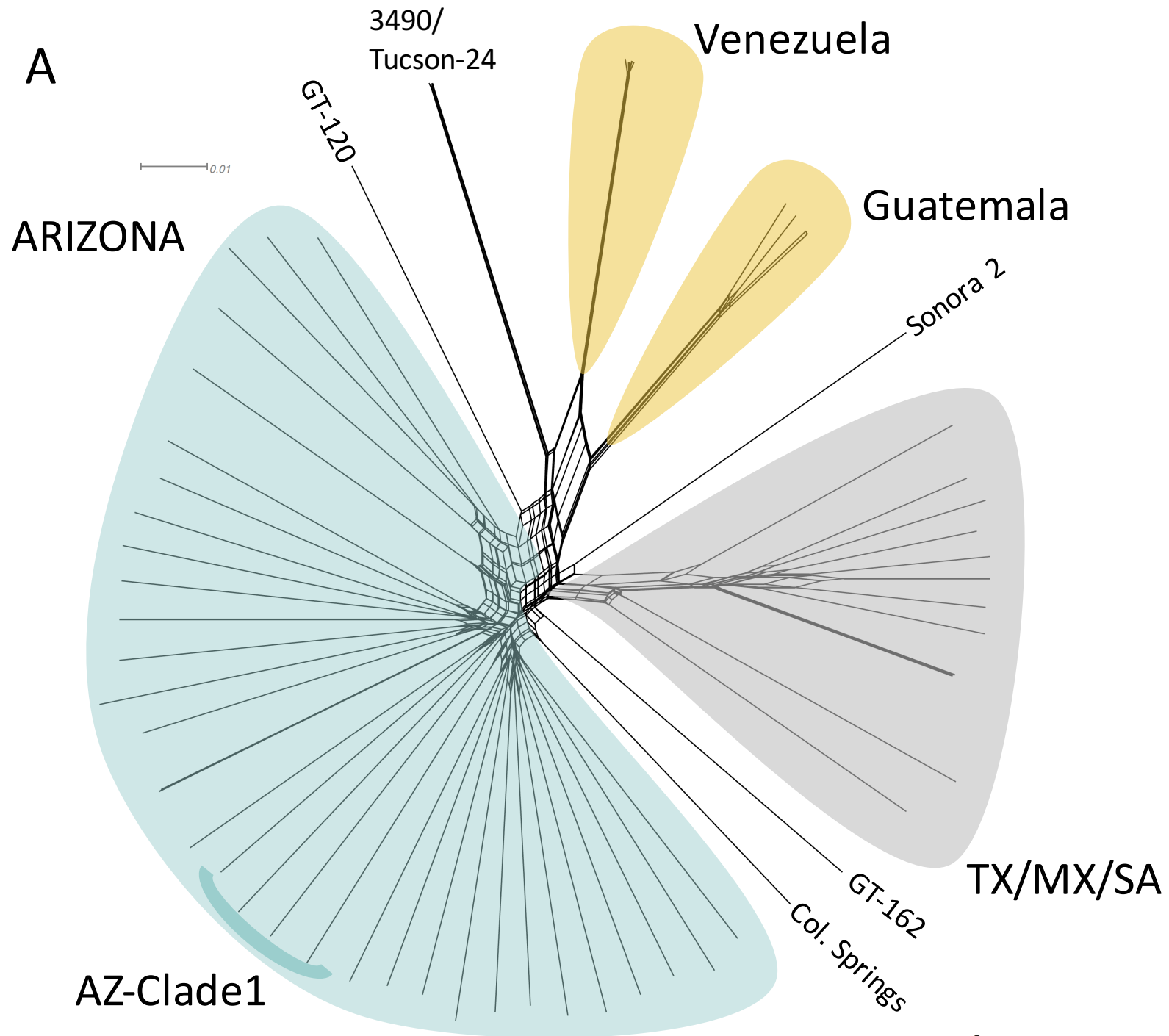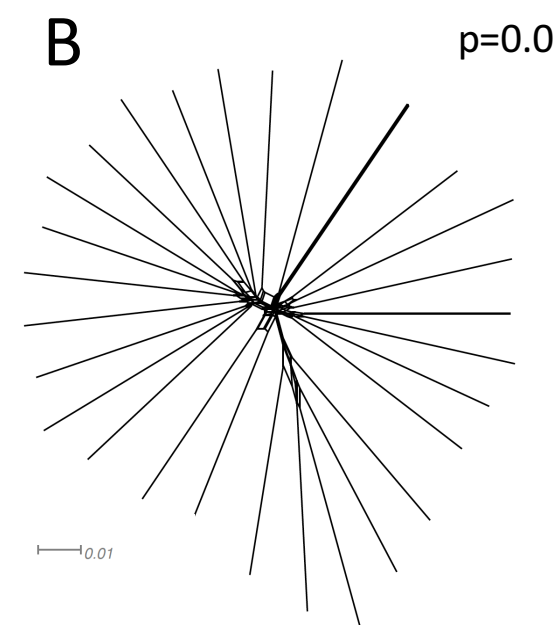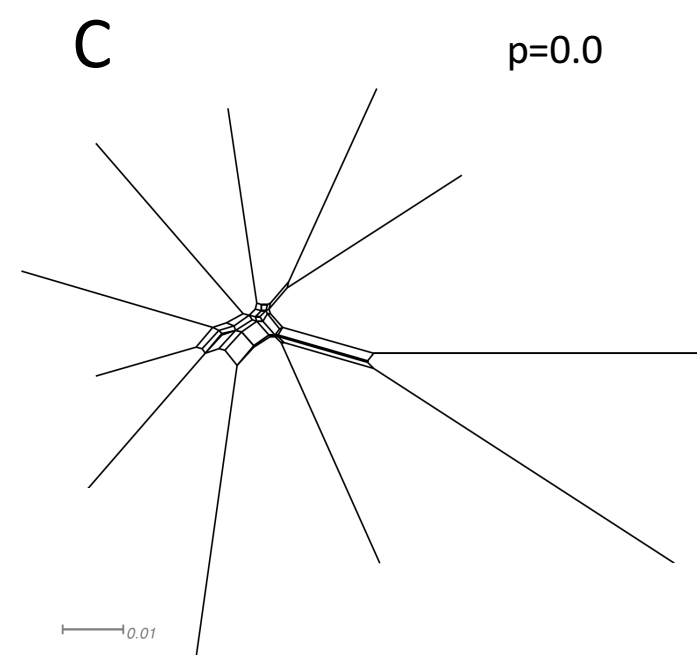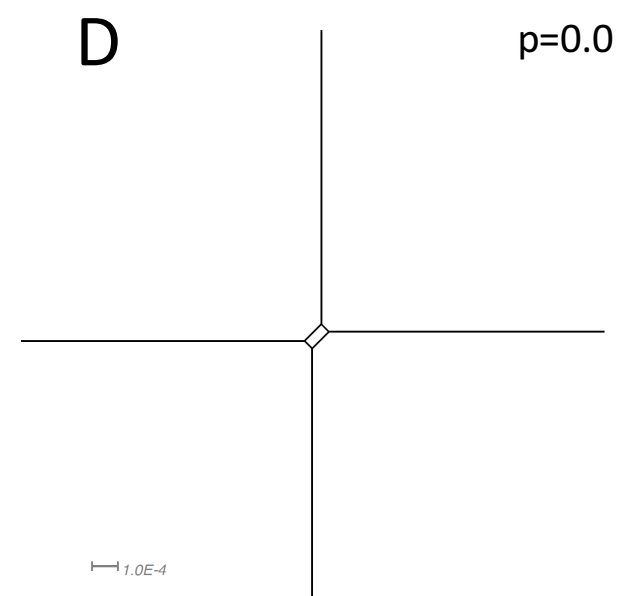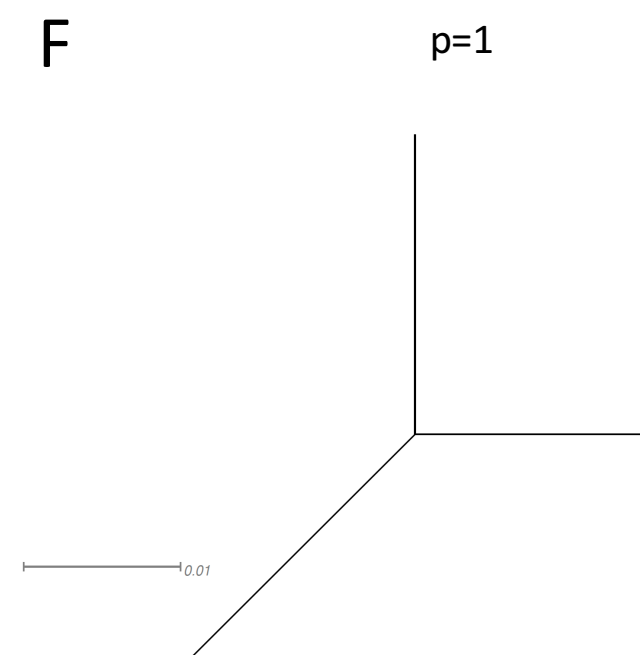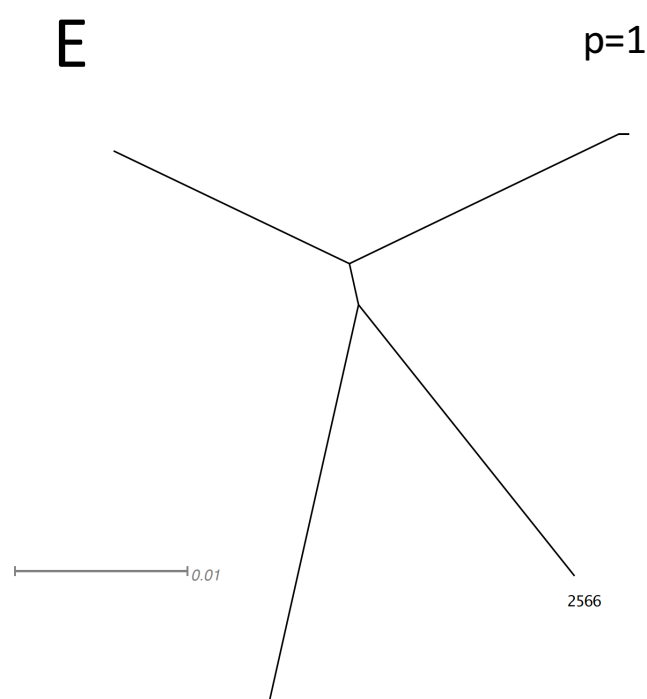

Supplement: FIG S1 [file mBio.01976-19-sf001.pdf]
