# Supplementary material for: Population Structure and Genetic Diversity among Isolates of Coccidioides posadasii in Venezuela and Surrounding Regions
Source: mBio. 2019 Nov 26;10(6):e01976-19. doi: 10.1128/mBio.01976-19 (PMC6879716; doi:10.1128/mBio.01976-19)
Supplement: TABLE S3 [file mBio.01976-19-st003.docx]

**Supplemental Data 3. Pairwise genetic distances.** We used asymptotic 2-sample Permutation Test to compare intra and interspecific differences.

|  | **C. immitis** | **AZ1** | **TXMXSA** | **Venez.** | **Guat.** | **GT162** | **Phoenix** | **TUC24** | **Tucson** |
| --- | --- | --- | --- | --- | --- | --- | --- | --- | --- |
| **C. immitis** | 0.02813333 | 6.293e-08 | < 2.2e-16 | < 2.2e-16 | < 2.2e-16 | < 2.2e-16 | < 2.2e-16 | < 2.2e-16 | < 2.2e-16 |
| **AZ1** | 0.9670682 | 0.05683333 | 1.843e-05 | 0.000333 | 1.34e-05 | 0.5714 | 0.1395 | 0.002165 | 0.02856 |
| **TXMX**  **SA** | 0.9678852 | 0.0646 | 0.04550909 | 3.775e-15 | 1.421e-14 | 1.462e-07 | < 2.2e-16 | 2.31e-12 | < 2.2e-16 |
| **Venez.** | 0.9682133 | 0.074375 | 0.07218182 | 0.0009047619 | 1.684e-06 | 0.04 | 7.629e-06 | 0.2367 | 2.328e-10 |
| **Guat.** | 0.9665909 | 0.0670625 | 0.06702564 | 0.054875 | 0.01466667 | 1.684e-06 | 0.0119 | 0.03571 | 2.2e-16 |
| **GT162** | 0.9660769 | 0.059 | 0.06641667 | 0.0725 | 0.068125 | 0.059 | 0.5294 | 0.4 | 0.2571 |
| **Phoenix** | 0.9663802 | 0.05853333 | 0.06716 | 0.07590323 | 0.06766667 | 0.0606875 | 0.05380303 | 2.2e-16 | 2.096e-13 |
| **Tuc24** | 0.9663957 | 0.06866667 | 0.07091667 | 0.0009047619 | 0.063 | 0.06575 | 0.07228571 | 0.055 | 3.057e-07 |
| **Tucson** | 0.9663957 | 0.06056 | 0.06772059 | 0.07581818 | 0.06805882 | 0.06223529 | 0.06044 | 0.06723529 | 0.05788235 |
